# Mechanistic effect of the human *GJB6* gene and its mutations in HaCaT cell proliferation and apoptosis

**DOI:** 10.1590/1414-431X20187560

**Published:** 2018-07-23

**Authors:** Yuting Lu, Ruili Zhang, Zhenying Wang, Shuhua Zhou, Yali Song, Lamei Chen, Nan Chen, Wenmin Liu, Canan Ji, Wangli Wu, Li Zhang

**Affiliations:** 1 Department of Dermatology, Huadu District People's Hospital of Guangzhou, Guangzhou, China; 2 Department of Dermatology, Shandong Provincial Hospital affiliated to Shandong University, Jinan, China; 3 Department of Dermatology, Weihai Municipal Hospital, Yantai, China

**Keywords:** Clouston syndrome, *GJB6* gene, Connexin 30, Caspase, Apoptosis

## Abstract

We constructed lentiviral vectors containing the human wild-type *GJB6* gene and the mutant variants *A88V* and *G11R*. The three proteins were stably expressed by the Tet-on system in the HaCaT cell line and used to study the functional effect of the variants. The CCK-8 assay and flow cytometric analyses were used to determine the levels of cell proliferation and apoptosis. Western blot analyses were performed to analyze the relevant clinical indicators of hidrotic ectodermal dysplasia and markers of apoptosis in transfected HaCaT cells. The CCK8 assay and the flow cytometry results showed a significant increase (P<0.05) in the apoptosis of HaCaT cells expressing the *A88V* and *G11R* mutants. In addition, we demonstrated that the *A88V* and *G11R* mutants induced the apoptosis of transfected HaCaT cells via the activation of caspase-3, -8, -9, and PARA. No change was observed in the activity of BAX compared with the control. This study provides further clarification on the mechanisms underlying the effect of the mutant variants *A88V* and *G11R* of the *GJB6* gene on the induction of HaCaT cell apoptosis.

## Introduction

Hidrotic ectodermal dysplasia (HED), also known as Clouston syndrome, is a rare autosomal dominant genetic skin disorder (1). It is characterized by alopecia, nail dystrophy, and palmoplantar hyperkeratosis (2). Mutations in the GJB6 gene cause HED, hereditary autosomal dominant non-syndromic deafness, and keratitis-ichthyosis-deafness syndrome. This gene encodes the gap junction protein connexin 30 (Cx30) (3). Cx30 is a member of the large gap-junction protein family, and it plays a role in the homeostasis of the epidermis and inner ear through gap junction intercellular communication. Lamartine et al. first confirmed that *GJB6* is the disease-causing gene of HED (4 [Bibr B05]). To date, five mutations have been found in patients with HED: *G11R*, *V37E*, *D50N*, *A88V*, and *N14S* in *GJB6* (3–6). We previously reported the *G11R* mutation in a Chinese family that caused HED of the hair and nails only (7).

In the process of our experiments, we found that the expression of *GJB6* mutants induced HatCaT cell death within 48 h (8). Berger et al. (9) determined that the *A88V* mutant caused cell death through membrane disruption within 24 h of expression. In this study, we investigated the mechanistic effects of the human *GJB6* gene and its mutants by constructing lentiviral vectors containing human wild-type *GJB6* and the variants *A88V* and *G11R*. We then stably expressed these proteins via the Tet-on system in the HaCaT cell line (10). This facilitated research on the mechanisms of apoptosis induction by the expression of these mutants in HaCaT cells.

## Material and Methods

### Cell culture

The HaCaT cell line and the Tet-on expression system were used to stably express vectors containing the wild-type *GJB6* gene and its mutant variants *A88V* and *G11R*. The vector inserts were obtained by whole gene synthesis. The sequences used were GJB6: NM_006783 (A88V) and GJB6: NM_006783 (G11R). The cells were cultured in RPMI 1640 medium (Gibco, USA) supplemented with 10% fetal bovine serum (Gibco) in a humidified incubator maintained at 37°C under 5% CO_2_. Once the cells had reached 80–100% confluence, the cells were detached using 0.25% trypsin-EDTA (Hyclone, USA) and then passaged.

### Cell proliferation

Cell proliferation was measured with the CCK8 assay (Dojindo, China). The HaCaT cells, which were transfected with either the negative control (NC) virus, the wild-type *GJB6* gene (WT), or the *A88V* and *G11R* mutants, were divided into two groups: 1) control group: NC, WT, A88V, and G11R cultured without doxycycline (free DOX); and 2) experimental group: NC, WT, A88V, and G11R cultured with DOX. The HaCaT cells were aliquoted into 96-well plates and incubated overnight to allow cell attachment. After 4, 8, 12, 24, 36, and 48 h of incubation following the addition of DOX or free DOX, the cells in each well were incubated with 10 μL of CCK8 for 1 h to measure the levels of cell proliferation.

### Flow cytometry analysis

FITC annexin V and propidium iodide (PI) staining were performed using the FITC annexin V Apoptosis Detection Kit from BD Biosciences (USA) to determine the level of apoptosis. The control and experimental group were first induced with DOX and free DOX, respectively. After 8 h, the cells were collected from the plates by trypsinization and washed twice with PBS. The cells were then resuspended in 100 μL binding buffer and 5 μL FITC annexin V and 5 μL PI were added to each well as per the manufacturer's instructions. After 15 min incubation in the dark, 400 μL binding buffer was added to the cells and the FITC annexin V/PI-stained cells were analyzed by flow cytometry. The percentages of apoptotic cells were measured with the BD FACSDiva 7.0 flow cytometer (BD Biosciences).

### Western blot analysis

Western blot analyses were performed to analyze the sequence-specific effect of Cx30 on the expression of apoptosis-related proteins in the transfected HaCaT cells. Briefly, 12 h post treatment, cells were lyzed in RIPA buffer (Solarbio, China), and proteins were separated by SDS-PAGE and transferred to microporous polyvinylidene difluoride (PVDF) membranes (Solarbio). The membranes were incubated in 5% Blotto non-fat dry milk (Santa Cruz Biotechnology, USA) with 0.05% Tween-20 (Sigma-Aldrich, USA) in PBS (PBS-T) for 1 h at room temperature and subsequently incubated overnight at 4°C with primary antibodies against Cx30 (1:1000, rabbit, Invitrogen, USA), BAX (1:1000, rabbit, Cell Signaling Technology, USA), caspase-3, cleaved caspase-3 (1:1000, rabbit, Cell Signaling Technology), and β-actin (1:1000, mouse, Sigma-Aldrich). Before incubation with HRP-conjugated goat anti-mouse or anti-rabbit secondary antibodies, the membranes were washed three times (10 min each) in PBS-T buffer. The blots were then scanned and densitometry measurements were carried out with the LAS 4000 gel-imaging system (Fujifilm, Japan). Each signal was normalized to the β-actin loading control in the same lane.

### Statistical analysis

Data are reported as means±SD. Statistical significance was defined as P<0.05 using Student's *t*-test.

## Results

### Changes in cell morphology after induction with doxycycline (DOX)

**Figure 1.**
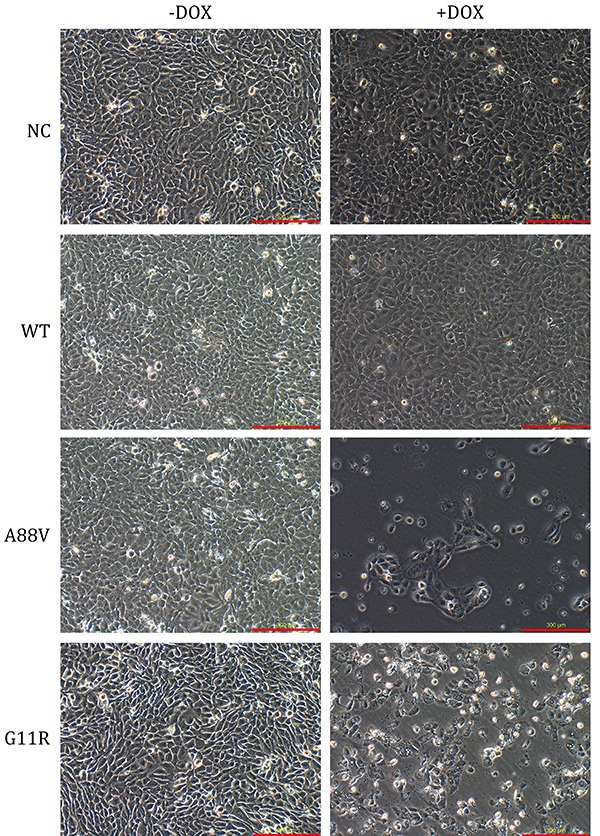
Morphology of cells transfected by negative control virus (NC) and cells expressing wild-type *GJB6* gene (WT) were not changed after the cells were induced by doxycycline (DOX). However, the morphology of cells expressing the *A88V* and *G11R* mutants became senescent with cell nucleus presenting pyknosis (100× magnification; bar: 300 μm).

Cellular morphological changes were observed under the microscope (100×) after induction by DOX. The morphology of cells transfected with the negative control virus or wild-type *GJB6* remained unchanged after the cells were induced. However, the morphology of cells expressing the *A88V* and *G11R* mutants became senescent and the cell nuclei underwent pyknosis. The cell morphology changes were exhibited by the mutant-expressing cells within 8 h of DOX induction and the cells were mostly dead after 48 h. The surviving cells after 48 h were senescent with pyknotic cell nuclei (Figure 1).

### Reduction of HaCaT cell viability in cells transfected with the *A88V* and *G11R* mutants

**Figure 2.**
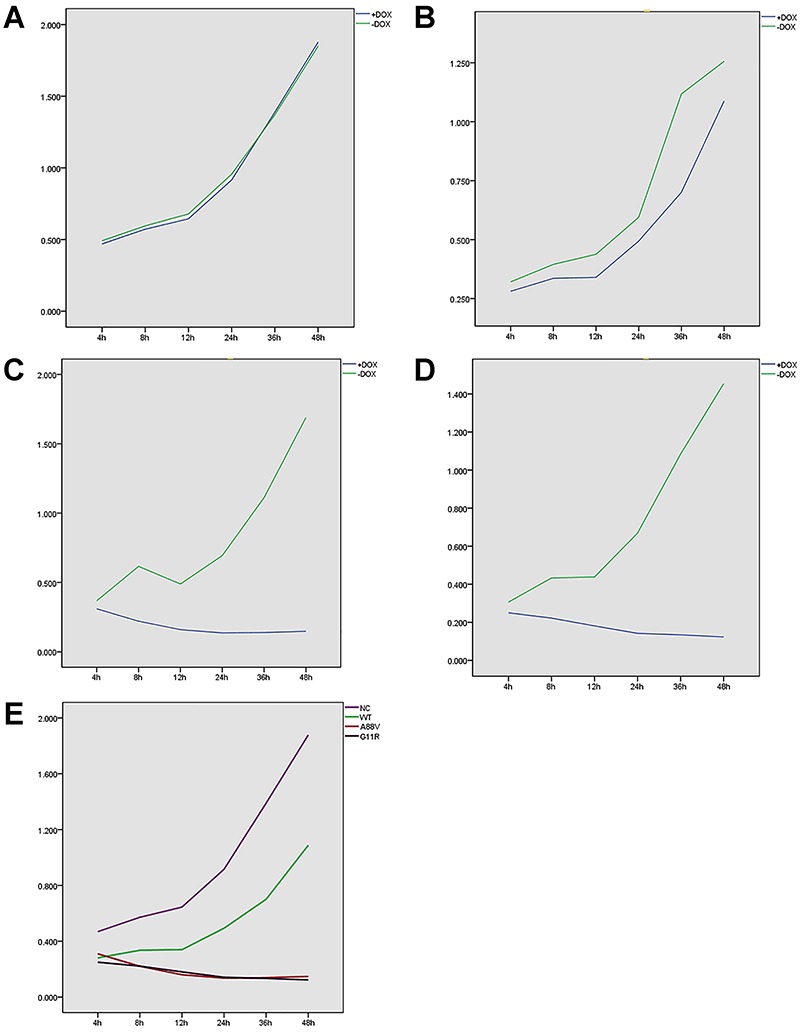
CCK8 test of cell proliferation showing absorbance values of four cell lines induced or not by doxycycline (+DOX, and -DOX): *A*, Negative control (NC); *B*, wild type (WT), *C*, *A88V* mutant, *D*, *G11R* mutant, and *E*, HaCat cells. Statistical analysis was done with the *t*-test.

We measured the absorbance of each group of cultured HaCaT cells with the CCK8 assay after 4, 8, 12, 24, 36, and 48 h incubation after induction (Table 1). The results indicated that the cell viability of HaCaT cells transfected with the negative control virus (NC) and induced by DOX were not significantly different from the NC cells that were not induced by DOX (P>0.05) (Figure 2A). Cells expressing the wild-type *GJB6* gene induced by DOX after 4, 8, 12, 24, and 36 h incubation were significantly more viable than the same cells not induced by DOX (P<0.05) (Figure 2B). In contrast, the DOX-induced cells expressing the *A88V* and *G11R* mutants after 4, 8, 12, 24, 36, and 48 h incubation were significantly less viable than the same cells not induced by DOX (P<0.05) (Figure 2C and 2D). Furthermore, the results of the LSD test showed that the DOX-induced cells expressing the *A88V* and *G11R* mutants were significantly less viable compared with the cells expressing the wild-type *GJB6* gene or the negative control virus across all time-points (Figure 2E). The CCK8-based cell proliferation assay showed no significant difference in proliferation between the G111R- and A88V-expressing cells, while these two groups of cells did show significantly reduced cell proliferation in comparison with the NC and wild-type groups.

**Table 1.** Cell growth inhibition evaluated by CCK8 analysis of HaCat cells induced or not by doxycycline (+DOX, and -DOX).

Group	4 h	8 h	12 h	24 h	36 h	48 h
NC						
+DOX	0.469±0.035	0.572±0.110	0.645±0.032	0.916±0.036	1.390±0.213	1.878±0.357
-DOX	0.492±0.018	0.596±0.070	0.679±0.149	0.957±0.093	1.370±0.238	1.850±0.287
t	1.683	0.554	0.667	1.249	0.184	0.180
P	0.112	0.587	0.514	0.230	0.856	0.860
WT						
+DOX	0.281±0.011	0.336±0.027	0.340±0.043	0.494±0.025	0.701±0.052	1.088±0.198
-DOX	0.321±0.014	0.395±0.022	0.438±0.065	0.595±0.011	1.118±0.186	1.256±0.213
t	6.630	5.141	4.644	10.665	6.462	1.729
P	**0.000**	**0.000**	**0.000**	**0.000**	**0.000**	0.103
*A88V*						
+DOX	0.310±0.007	0.221±0.021	0.160±0.014	0.136±0.002	0.139±0.004	0.148±0.009
-DOX	0.368±0.029	0.616±0.081	0.489±0.042	0.694±0.034	1.124±0.097	1.690±0.172
t	5.809	14.231	22.142	48.198	30.444	26.845
P	**0.000**	**0.000**	**0.000**	**0.000**	**0.000**	**0.000**
*G11R*						
+DOX	0.250±0.013	0.222±0.021	0.181±0.018	0.142±0.002	0.134±0.003	0.123±0.003
-DOX	0.306±0.017	0.433±0.031	0.438±0.053	0.669±0.038	1.084±0.090	1.454±0.220
t	7.769	17.064	13.875	41.827	31.781	18.127
P	**0.000**	**0.000**	**0.000**	**0.000**	**0.000**	**0.000**

Data are reported as means±SD absorbance values for n=9. NC: Negative control; WT: wild type; A88V: *A88V* mutant; G11R: *G11R* mutant. Statistical analysis was done with the *t*-test.

### Apoptosis induction by the *A88V* and *G11R* mutations of the *GJB6* gene

**Figure 3.**
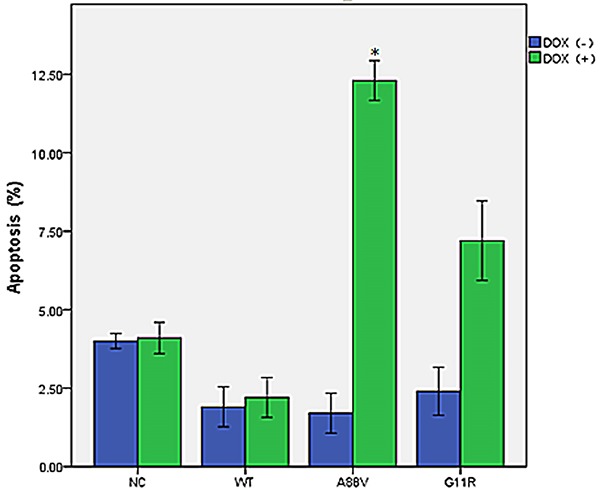
Flow cytometry results showing levels of apoptotic cells in negative control (NT), wild type (WT), *A88V* mutant, and *G11R* mutant cells, induced or not by doxycycline (+DOX, and -DOX). Data are reported as means±SD. *P<0.05, *t*-test.

To determine whether the growth inhibitory effect of the *A88V* and *G11R* mutants of the *GJB6* gene is associated with apoptosis, annexin V/PI staining of HaCaT cells expressing the wild-type *GJB6* gene and the *A88V* and *G11R* mutants were analyzed with flow cytometry (Figure 3). The cells were classified as Q2 necrotic cells (annexin V+, PI+), Q3 healthy cells (annexin V−, PI−) and Q4 apoptotic cells (annexin V+, PI−). The HaCaT cells expressing the *A88V* and *G11R* mutants showed increased apoptosis and lower cell viability that was significantly different (P<0.05) than the cells transfected with the negative control virus or the wild-type *GJB6* gene.

### Induction of protein cleavage by the *A88V* and *G11R* mutants

Expression of the *A88V* and *G11R* mutants resulted in elevated levels of the cleaved forms of caspase-3, caspase-8, caspase-9, and PARP, which are all markers of apoptotic activation while the same markers were unchanged in cells transfected with the negative control virus or the wild-type *GJB6* gene. The level of BAX remained unchanged in all groups.

**Figure 4.**
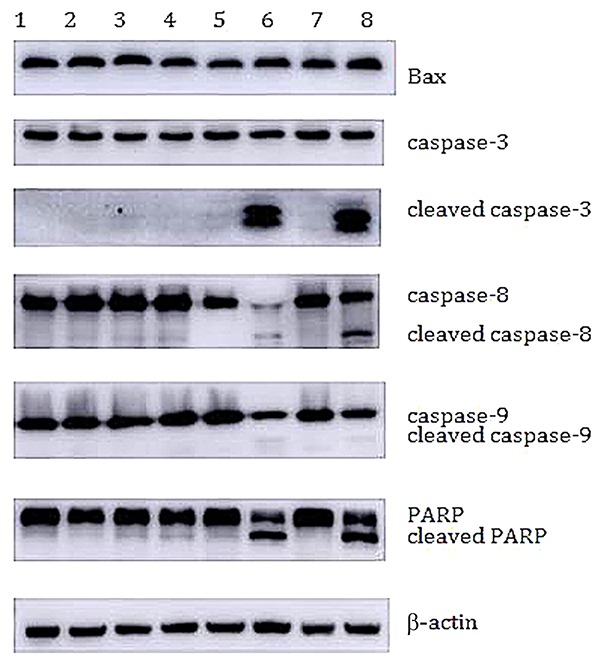
Effects of the *A88V* and *G11R* mutants on apoptosis-related proteins in HaCat cells assessed by western blotting analysis. +/−DOX: with or without doxycycline. *Lanes*: *1*: NC(−DOX); *2*: NC(+DOX); *3*: WT(−DOX); *4*: WT(+DOX); *5*: A88V(−DOX); *6*: A88V(+DOX); *7*: G11R(−DOX); *8*: G11R(+DOX).

Figure 4 shows the effects of the *A88V* and *G11R* mutations of *GJB6* on apoptosis-related proteins.

## Discussion

In the present study, we discovered that the *A88V* and *G11R* mutants induced apoptosis of HaCaT cells, which quickly led to population-wide cell death. To further research the mechanistic effect of the human *GJB6* gene and mutations on the proliferation and apoptosis of HaCaT cells, we constructed lentiviral vectors containing the human *GJB6* gene and the mutants, and stably expressed these proteins using the Tet-on system in the HaCaT cell line (10). The Tet-on system has potential for numerous applications, including the study of gene function and gene therapy. This system enables the efficient expression of genes following induction by DOX (11). It also mitigates the poor regulation of gene expression of the many other expression systems, as our team experienced when we first constructed the traditional overexpression lentivirus vector (8).

Apoptosis is a conserved phenomenon that plays a critical role in the regulation of the cellular activities of eukaryotes, and the process is characterized by chromatin condensation (12,13). In our study, the HaCaT cells expressing the *A88V* and *G11R* mutants were found to exhibit significantly increased apoptosis. Moreover, the cells stained with annexin V and PI indicate apoptotic cells (annexin V) and dead or late apoptotic cells (PI). Early apoptotic cells are PI-negative and annexin V-positive, while dead or apoptotic cells are positive for both annexin V and PI. Based on the cell staining results, the *A88V* and *G11R* mutants clearly increased apoptosis in HaCaT cells.

There are two major apoptotic pathways, classified as extrinsic or intrinsic cell death (13,14). The results of the present study indicated that the *A88V* and *G11R* mutants induced the activation of caspase-3, caspase-8, caspase-9, and PARP. However, there was no change in BAX activity compared with the control indicating that this protein may play a minor role at most in the apoptosis of HaCaT cells (15). Much evidence suggests that the apoptotic process is triggered by the activation of caspases in various cell types (16).

Caspase, a cysteinyl aspartate-specific proteinase, is an important player in cell apoptosis. Caspases are divided into two broad categories: the initiator caspases and the effector caspases (17,18). Apoptotic initiators (such as caspase-2, -8, -9, and -10) activate the downstream cascade of necroptosis with the help of the adapter proteins. Active caspase-8 then either initiates apoptosis directly by cleaving and thereby activating executioner caspases, or by activating the intrinsic apoptotic pathway through the cleavage of BID to induce efficient cell death (19,20). Cascade-initiating pathways converge on the activation of the downstream effector caspases (such as caspase-3, -6, and -7) that act to kill the cells by cleaving death substrates.

As previously reported, caspase-3 is a member of the activated-caspase family (21). It is the major activated factor in the process of apoptosis, and its activation is a sign that apoptosis has entered the irreversible phase. This protein is therefore considered the end executor of apoptosis. Usually, the extrinsic apoptosis pathway is activated through the binding of a ligand to a death receptor, which is mediated by caspase-8, while the intrinsic apoptotic pathway is mediated by mitochondrial cytochrome c and caspase-9. Caspase is synthesized as an inactive proenzyme (zymogen) in living cells. The proenzyme is activated through cleavage, and this active form is involved in nuclear condensation, cellular detachment, and phosphatidylserine externalization, all hallmark characteristics of apoptosis. These effector caspases then cleave PARP (12), and the activated PARP confers apoptotic cells with their classic morphological and biochemical characteristics.

In summary, our results showed that the *A88V* and *G11R* mutants of *GJB6* may activate the downstream execution factor, caspase-3, both through the extrinsic apoptotic pathway mediated by caspase-8 and the intrinsic apoptotic pathway mediated by caspase-9. This rapidly induced HaCaT cell apoptosis by means of the lyase, PARP. Further investigations are required to determine whether the *A88V* and *G11R* variants can also induce apoptosis through pathways other than those mediated by caspases, and to determine how these mutants lead to the clinical manifestations of HED.
